# Genetic Variants in Double-Strand Break Repair Pathway Genes to Predict Platinum-Based Chemotherapy Prognosis in Patients With Lung Cancer

**DOI:** 10.3389/fphar.2022.915822

**Published:** 2022-07-11

**Authors:** Jun-Yan Liu, Ting Zou, Ji-Ye Yin, Zhan Wang, Chong Liu, Han-Xue Huang, Fei-Xiang Ding, Meng-Rong Lei, Ying Wang, Min Liu, Zhao-Qian Liu, Li-Ming Tan, Juan Chen

**Affiliations:** ^1^ Department of Orthopaedics, Xiangya Hospital, Central South University, Changsha, China; ^2^ National Institution of Drug Clinical Trial, Xiangya Hospital, Central South University, Changsha, China; ^3^ Departments of Clinical Pharmacology, Xinagya Hospital, Central South University, Changsha, China; ^4^ Institute of Clinical Pharmacology and Hunan Key Laboratory of Pharmacogenetics, Central South University, Changsha, China; ^5^ Lung Cancer and Gastrointestinal Unit, Department of Medical Oncology, Hunan Cancer Hospital, Affiliated Cancer Hospital of Xiangya School of Medicine, Changsha, China; ^6^ Hunan Clinical Research Center in Gynecologic Cancer, Hunan Cancer Hospital, Affiliated Cancer Hospital of Xiangya School of Medicine, Changsha, China; ^7^ Xiangya School of Pharmaceutical Sciences, Central South University, Changsha, China; ^8^ Department of Pharmacy, The Second People’s Hospital of Huaihua City, Huaihua, China; ^9^ Department of Pharmacy, Xinagya Hospital, Central South University, Changsha, China; ^10^ National Clinical Research Center for Geriatric Disorders, Xiangya Hospital, Central South University, Changsha, China

**Keywords:** lung cancer, platinum-based chemotherapy, prognosis, genetic polymorphisms, MAD2L2, TNFRSF1A

## Abstract

**Objective:** The purpose of this study was to investigate the associations of genetic variants in double-strand break (DSB) repair pathway genes with prognosis in patients with lung cancer treated with platinum-based chemotherapy.

**Methods:** Three hundred ninety-nine patients with lung cancer who received platinum-based chemotherapy for at least two cycles were included in this study. A total of 35 single nucleotide polymorphisms (SNPs) in DSB repair, base excision repair (BER), and nucleotide excision repair (NER) repair pathway genes were genotyped, and were used to evaluate the overall survival (OS) and the progression-free survival (PFS) of patients who received platinum-based chemotherapy using Cox proportional hazard models.

**Results:** The PFS of patients who carried the *MAD2L2* rs746218 GG genotype was shorter than that in patients with the AG or AA genotypes (recessive model: *p* = 0.039, OR = 5.31, 95% CI = 1.09–25.93). Patients with the TT or GT genotypes of *TNFRSF1A* rs4149570 had shorter OS times than those with the GG genotype (dominant model: *p* = 0.030, OR = 0.57, 95% CI = 0.34–0.95). We also investigated the influence of age, gender, histology, smoking, stage, and metastasis in association between SNPs and OS or PFS in patients with lung cancer. DNA repair gene SNPs were significantly associated with PFS and OS in the subgroup analyses.

**Conclusion:** Our study showed that variants in *MAD2L2* rs746218 and *TNFRSF1A* rs4149570 were associated with shorter PFS or OS in patients with lung cancer who received platinum-based chemotherapy. These variants may be novel biomarkers for the prediction of prognosis of patients with lung cancer who receive platinum-based chemotherapy.

## Introduction

Lung cancer has one of the highest rates of cancer-related mortality (Parkin et al., 1999; Siegel et al., 2021). Approximately 2.2 million new lung cancer cases and 1.8 million deaths resulting from lung cancer were reported in 2020, which was double the number reported 30 years earlier (Parkin et al., 1999; Siegel et al., 2021). The overall 5-year survival rate for lung cancer is less than 18% due to rapid progression and late-stage diagnosis (Alam et al., 2020). Lung cancer mainly consists of non–small cell lung cancer (NSCLC) and small cell lung cancer (SCLC), which occur in 85:15 ratio (Shi and Sun, 2015; Schwartz and Cote, 2016). Surgery, radiotherapy, chemotherapy, immunotherapy, and targeted therapy are the primary approaches for the treatment of lung cancer. Specific management is contingent on staging and pathohistological type (Kalemkerian et al., 2018; Ettinger et al., 2019). Development of targeted therapies and immunotherapy has resulted in substantial clinical benefits. However, the majority of patients do not have activating mutations and do not experience long-term stable remission (Hirsch et al., 2017; Arbour and Riely, 2019). Chemotherapy is the main treatment for lung cancer, with platinum-based chemotherapy the most widely-used approach.

Platinum-based chemotherapy has been widely used as a therapeutic regimen to treat cancer, including patients with lung cancer, since the first platinum agent, cisplatin, was approved over 40 years ago (Rottenberg et al., 2021). Cisplatin, carboplatin, and oxaliplatin are the three main platinum-based antineoplastic drugs (Low et al., 2021). Cisplatin is the standard treatment for NSCLC, and is the first choice to treat patients with advanced cancers without treatable gene mutations (Kryczka et al., 2021). The platinum-doublet chemotherapy, platinum combined with etoposide, is recommended as a first-line treatment for late-stage SCLC (Thai et al., 2021; Zugazagoitia and Paz-Ares, 2022). Although platinum-based chemotherapy can improve survival rate, patients treated with platinum-based drugs often suffered from drug resistance, resulting in poor prognosis and therapeutic failure. There are many prognostic factors such as genetic polymorphisms, age, gender, histology type, smoking, metastasis, and clinical stage that have been reported to be connected to platinum-based chemotherapy sensitivity (Cescon et al., 2015; Chen et al., 2016; Yin et al., 2016). Therapeutic efficacy is unsatisfactory and unpredictable. Therefore, the identification of novel biomarkers may help to identify therapeutic avenues that can improve survival time.

Investigation of mechanisms of drug resistance is of great importance for the improvement of the prognosis of lung cancer in response to platinum-based chemotherapy. Given that platinum-based drugs induce DNA fragmentation through crosslinking with DNA to form DNA adducts, alteration of DNA repair mechanisms can affect tumor sensitivity to platinum drugs (Rottenberg et al., 2021). Base excision repair (BER), nucleotide excision repair (NER), DNA mismatch repair (MMR), homologous recombination (HR), and non-homologous end joining (NHEJ) are the major DNA repair pathways, among which HR and NHEJ are responsible for repairing double-strand breaks (DSBs). Furthermore, NHEJ plays an important role in the DNA damage response system (Gupta et al., 2018; Xu and Xu, 2020; Jiang et al., 2021), which can directly link the ends of DSBs by DNA ligase to promote ligation of DNA ends. This process is characterized by impedance of homologous DNA sequences, while HR uses the intact sister chromatid as a template (Huang and Dynan, 2002; Andres et al., 2015; Xing et al., 2015; Almohaini et al., 2016; Kulkarni et al., 2016; Menon and Povirk, 2017; Reid et al., 2017). Previous studies have shown the importance of double-strand break repair (DSBR) pathways in platinum chemotherapy resistance (Kryczka et al., 2021). A numbers of DNA repair genes have been confirmed to be related to platinum-based chemotherapy resistance in patients with lung cancer, including the *XRCC5* and *HSPB1* genes. However, few studies have focused on polymorphisms in the DSBR pathway genes.

We investigated the associations between SNPs in the *MAD2L2*, *XPC*, *XRCC3*, *BRCA2*, *RAD52*, *NFKB1*, *NFKBIA*, *TNFRSF1A*, or *FASN* genes and prognosis in patients with lung cancer who received platinum-based chemotherapy.

## Patients and Methods

### Patients and Data Collection

The inclusion criteria for 399 patients with lung cancer were as follows: 1) all patients attended at the Xiangya Hospital of Central South University or Affiliated Cancer Hospital of Xiangya School of Medicine (Changsha, Hunan, China) from August 2009 to May 2013; 2) patients with lung cancer received platinum-based chemotherapy for at least two cycles; 3) patients with lung cancer had not undergone surgery, radiotherapy, targeted drug therapy, or other biological therapy before chemotherapy. The research proposal was approved by the Ethics Committee of Xiangya Hospital, Central South University. All patients provided written informed consent prior to participating in the study.

The deadline for patient enrollment was 15 July 2019. Standard follow-up clinical data included age, gender, smoking history, histology classification, TNM stage, and metastasis. The two main data processing approaches were PFS, which was defined as the time period from diagnosis until disease progression. Patients without OS and PFS data were removed from the study at the final follow-up. The overall survival was calculated as the time between lung cancer diagnosis and follow-up or death.

### Single Nucleotide Polymorphism Selection, DNA Extraction, and Genotyping

The SNPs genotyped in our study were *ERCC1* SNPs (rs2298881), *ERCC2* SNPs (rs1052555, rs238406), *ERCC4* SNP (rs1799801), *ERCC6* SNPs (rs2228527, rs3793784), *XPC* SNPs (rs2228000, rs2228001), *XRCC1* SNP (rs25489), *XRCC3* SNPs (rs1799794, rs861539), *BRCA1* SNP (rs799917), *BRCA2* SNPs (rs543304,rs206118), *RAD51* SNPs (rs12593359, rs1801320, rs1801321), *RAD52* SNPs (rs1051669, rs7963551), *POLH/POLR1C* SNP (rs6941583), *MAD2L2* SNPs (rs2233004, rs746218, rs2233006), *NFKB1* SNPs (rs230529, rs1585215, rs4648068), *NFKBIA* SNP (rs2233406), *TNF* SNP (rs1800629), *TNFRSF1A* SNPs (rs4149570, rs2234649), *TNFRSF1B* SNP (rs1061622), and *FASN* SNPs (rs1140616, rs2228309, rs4246445, rs4485435). Haploview was used to choose pair-wise tagging SNPs with pair wise *r* ^2^ threshold ≥0.8, and all SNPs had a minor allele frequency (MAF) greater than 0.05 (Table 1).

**TABLE 1:** 35 gene polymorphisms examined in this study.

Gene	SNP	Alleles	Call Rate (%)	MAF
ERCC1	rs2298881	C/A,G,T	96.24	0.402
ERCC2	rs1052555	G/A	100	0.143
	rs238406	T/G	96.24	0.471
ERCC4	rs1799801	T/C	98.50	0.298
ERCC6	rs2228527	T/A,C	100	0.118
	rs3793784	G/C	98.25	0.337
XPC	rs2228000	G/A	96.24	0.340
	rs2228001	G/C,T	98.50	0.411
XRCC1	rs25489	C/A,G,T	99.75	0.172
XRCC3	rs1799794	T/C	97.24	0.470
	rs861539	G/A	99.50	0.098
BRCA1	rs799917	G/A,C,T	97.49	0.423
BRCA2	rs543304	T/C,G	96.74	0.240
BRCA2	rs206118	A/C,G	98.25	0.246
RAD51	rs12593359	T/A,C,G	97.74	0.266
	rs1801320	G/C	96.49	0.226
	rs1801321	G/C,T	97.24	0.124
RAD52	rs1051669	C/T	96.24	0.245
	rs7963551	T/G	99.75	0.273
POLH	rs6941583	A/T	99.75	0.079
MAD2L2	rs2233004	G/A	99.25	0.090
	rs746218	G/A	92.48	0.211
	rs2233006	T/A	94.99	0.447
NFKB1	rs230529	A/G	95.99	0.491
	rs1585215	A/G	95.99	0.420
	rs4648068	A/G	99.75	0.474
NFKBIA	rs2233406	C/T	99.75	0.196
TNF	rs1800629	G/A	95.74	0.102
TNFRSF1A	rs4149570	T/G	95.74	0.487
	rs2234649	A/C	99.50	0.147
TNFRSF1B	rs1061622	T/G	99.50	0.247
FASN	rs1140616	C/T	97.49	0.372
	rs2228309	T/C	96.74	0.246
	rs4246445	A/G	97.99	0.453
	rs4485435	G/C	99.25	0.216

MAF, minor allele frequency.

All blood samples were collected and stored in EDTA tubes. We used a genomic DNA Purification Kit (Promega) to extract genomic DNA. Genotyping of all SNPs was performed using the Sequenom MassARRAY Genotyping Platform (Sequenom, San Diego, CA, United States).

### Statistical Analysis

Logistic regression was used to select covariates using the Cox proportional hazard model. The covariates included age, gender, histologic type, smoking status, clinical stage, and metastasis status. Three analysis models (additive model: compares major allele homozygotes versus heterozygotes versus minor allele homozygotes; dominant model: compares major allele homozygous versus combined heterozygotes and minor allele homozygous groups; recessive model: compares major allele-carrying genotypes with homozygous variant genotype) were used to calculate the associations between SNPs and prognosis. In the association analyses, we divided the patients into two or three groups by their genotypes of the SNPs. In additive model and dominant model, patients with wild type were used as a control group; and in recessive model, patients with wild type and heterozygote were used as a control group. The Cox proportional hazard regression analysis was used to analyze OS and PFS. All data were analyzed using SPSS 18.0 software (SPSS Inc., Chicago, IL, United States), PLINK (version 1.9, http://pngu.mgh.harvard.edu/purcell/plink/), and R 4.1.0. The associations between PFS or OS and SNPs were calculated as odds ratio (OR) and their 95% confidence intervals (95% CI) using unconditional logistic regression.

## Results

### Demographic Characteristics of Patient Characteristics

Three hundred ninety-nine patients with lung cancer were enrolled in this study. All included patients had received platinum-based chemotherapy as the first-line treatment. The patients were 21–75 years old, with a median age of 56 years old. In this study, 317 (79.4%) patients were male and 82 (20.6%) were female. Furthermore, 152 (38.1%) patients were non-smokers and 247 (61.9%) were smokers. In addition, 311 (77.9%) patients had NSCLC and 88 (22.1%) had SCLC. Finally, 351 (88.0%) patients were at advanced stages (stage Ⅲ/Ⅳ/ED), and the remaining 48 (12.0%) were at early stages (stage I/II/LD) (Table 2).

**TABLE 2:** Distribution of characteristics in patients with patients with lung cancer and prognosis analysis.

Variable	Patients (N%)	Death (N%)	MST-OS (year)	MST-PFS (year)
Age				
≤55	197 (49.4)	153 (77.6)	3.75	2.94
>55	202 (50.6)	168 (83.2)	4.67	4.37
Gender				
Male	317 (79.4)	256 (80.8)	4.39	3.87
Female	82 (20.6)	65 (79.3)	4.11	3.21
Histology				
NSCLC	311 (77.9)	256 (82.3)	4.34	3.26
SCLC	88 (22.1)	65 (73.9)	4.32	4.48
Smoking status				
Non-smoker	152 (38.1)	120 (78.9)	4.02	3.12
Smoker	247 (61.9)	200 (81.0)	4.45	3.91
Stage				
I/II/LD	48 (12.0)	36 (75.0)	5.00	4.38
III/IV/ED	351 (88.0)	281 (80.1)	4.31	3.34

MST, median survival time; LD, limitation disease; ED, extensive disease.

### Association Between *MAD2L2* rs746218 and PFS in Patients With Lung Cancer

**FIGURE 1:**
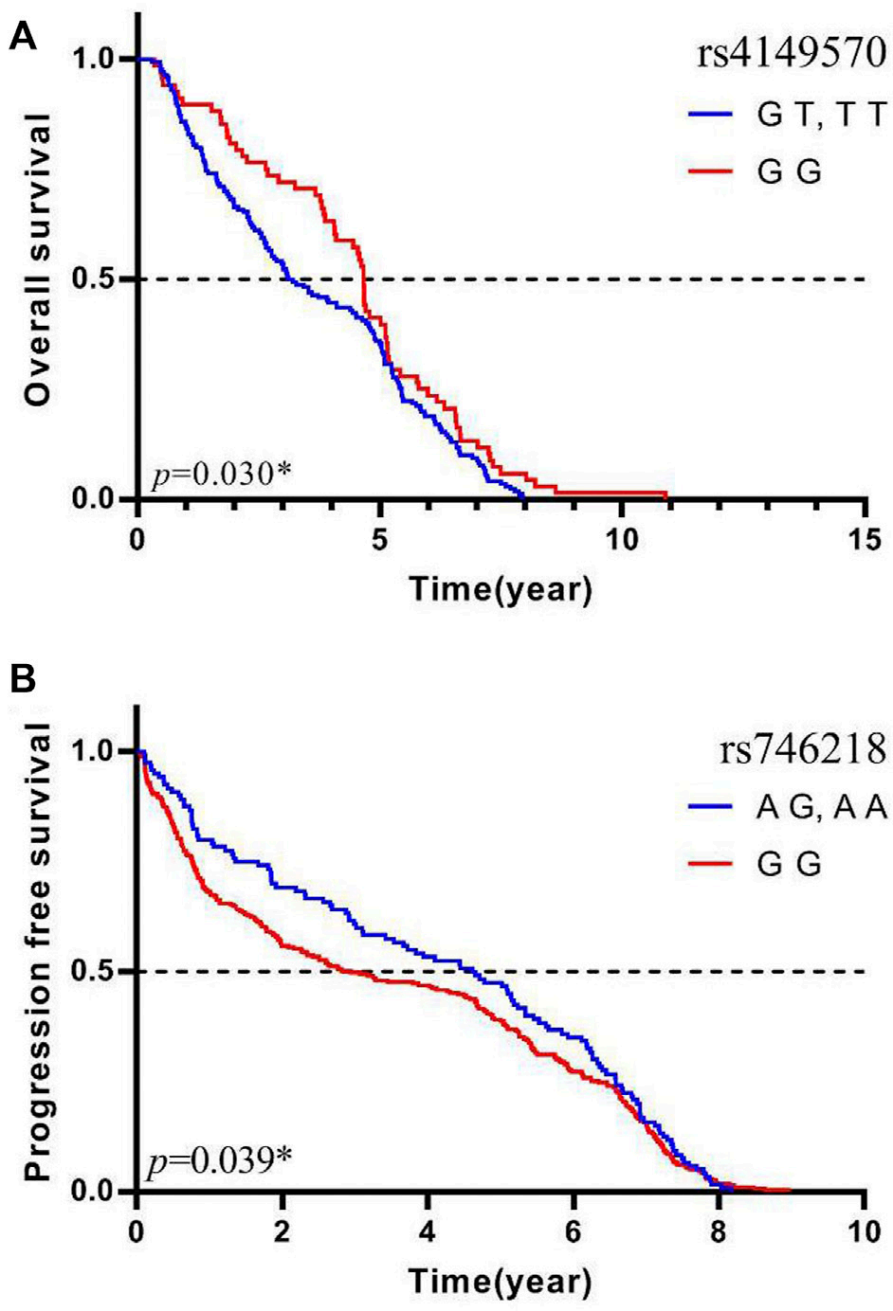
*MAD2L2* rs746218 and *TNFRSF1A* rs4149570 were significantly associated with platinum-based chemotherapy prognosis in patients with lung cancer. **(A)** *TNFRSF1A* rs4149570 was significantly associated with OS. **(B)** *MAD2L2* rs746218 was significantly associated with PFS.

Multivariate Cox regression adjusted for age, gender, histology type, smoking status, stage, and metastasis showed that the *MAD2L2* rs746218 polymorphism was significantly related to PFS in patients with lung cancer in the recessive model (*p* = 0.039, OR = 5.31, and 95% CI = 1.09–25.93) (Table 3). Patients carrying the AG or AA genotype had a significantly longer PFS times than those carrying the GG genotype (Figure 1A). Compared with other SNPs, *MAD2L2* rs746218 was significantly associated with PFS in the recessive model analysis in patients with lung cancer who received platinum-based chemotherapy.

**TABLE 3:** Association of the *MAD2L2* rs746218 polymorphisms and PFS in lung cancer patients.

Gene	Polymorphisms	Genotypes	MPFS (year)	Additive		Dominant		Recessive	
				OR (95%CI)	*p* value	OR (95%CI)	*p* value	OR (95%CI)	*p* value
MAD2L2	rs746218	GG	5.84	1.26 (0.84–1.90)	0.263	1.12 (0.7–1.78)	0.633	5.31 (1.09–25.9)	0.039*
		GA	3.67						
		AA	3.25						

MPFS, median progression-free survival; OR, odds ratio; CI, confidence interval; additive model: comparison between minor allele subjects and major allele subjects. Dominant model: comparison between minor allele carriers and major homozygous subjects. Recessive model: comparison between major allele carriers and minor homozygous subjects.

### Association Between *TNFRSF1A* rs4149570 and OS in Patients With Lung Cancer

Univariate Cox regression analysis was used to evaluate OS, and the results were adjusted for age, gender, histology type, smoking status, stage, and metastasis status. As shown in Table 4, *TNFRSF1A* rs4149570 was associated with OS in patients with lung cancer in the dominant model (*p* = 0.030, OR = 0.57, and 95% CI = 0.34–0.95). In the dominant model, the OS of patients who carried the rs4149570 GG genotype was significantly longer than that of patients carrying the TT or GT genotypes (Figure 1B). Compared with other SNPs, *TNFRSF1A* rs4149570 was most significantly associated with OS in the dominant model in patients with lung cancer who received platinum-based chemotherapy.

**TABLE 4:** Association of the *TNFRSF1A* rs4149570 polymorphisms and OS in lung cancer patients.

Gene	Polymorphisms	Genotype	MST (year)	Additive		Dominant		Recessive	
				OR (95%CI)	*p* value	OR (95%CI)	*p* value	OR (95%CI)	*p* value
TNFRSF1A	rs4149570	TT	5.32	0.74 (0.53–1.04)	0.084	0.57 (0.34–0.95)	0.030*	0.86 (0.48–1.52)	0.594
		TG	3.53						
		GG	4.66						

MST, median survival time; OR, odds ratio; CI, confidence interval; Additive model: comparison between minor allele subjects and major allele subjects, Dominant model: comparison between minor allele carriers and major homozygous subjects, Recessive model: comparison between major allele carriers and minor homozygous subjects.

### Stratification Analyses

**FIGURE 2:**
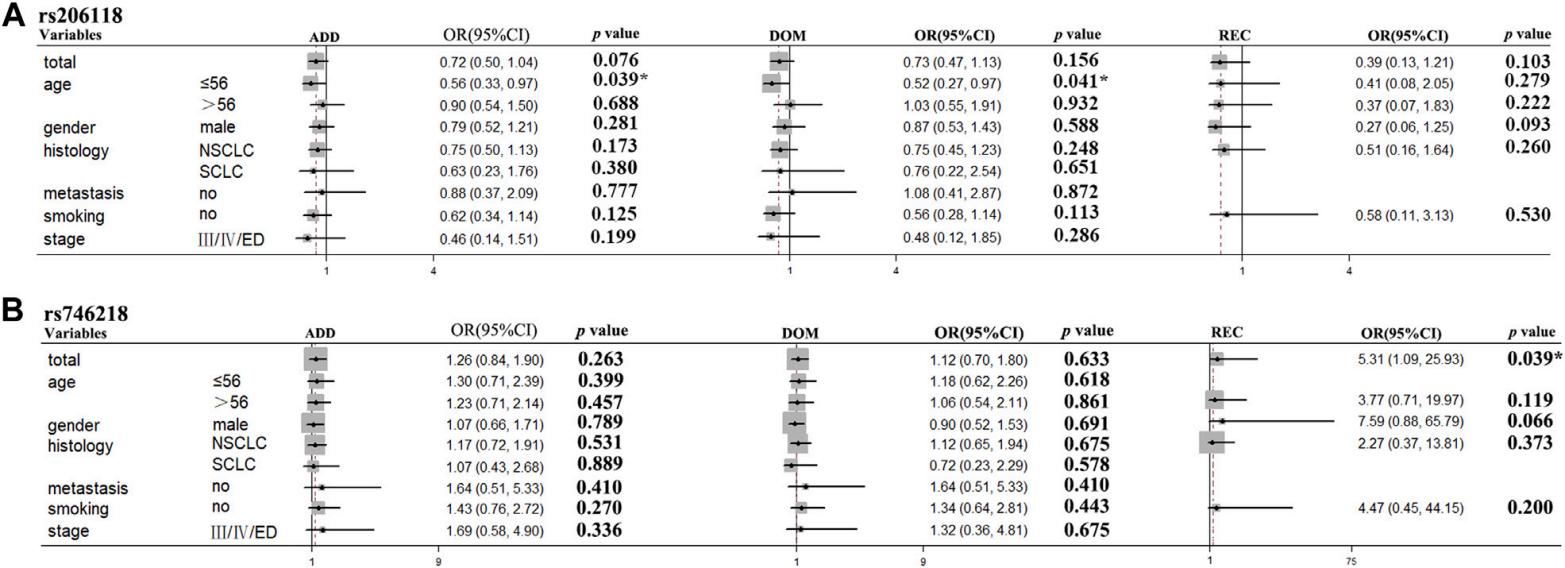
*BRCA2* rs206118 and *MAD2L2* rs746218 polymorphisms were significantly associated with survival time in the subgroups of patients with lung cancer treated with platinum-based chemotherapy. **(A)** *BRCA2* rs206118 polymorphism was significantly associated with PFS time in patients less than 55 years of age. **(B)** *MAD2L2* rs746218 polymorphism was significantly associated with PFS time in patients treated with platinum-based chemotherapy.

In the stratification analyses, age (≤56, >56), smoking status (no, yes), gender (male, female), histological type (NSCLC, SCLC), metastasis (no, yes), and Stage (I/II/LD, III/IV/ED) were evaluated as covariates for associations between SNPs and PFS. The following SNPs were significantly associated with PFS: *BRCA2* rs206118 in patients ≤56 years old (additive model: *p* = 0.039, OR = 0.56, and 95% CI = 0.33–0.97; dominant model: *p* = 0.041, OR = 0.52, and 95% CI = 0.27–0.97); *XRCC3* rs1799794 in patients >56 years old (dominant model *p* = 0.036, OR = 2.03, and 95% CI = 1.05–3.92); *NFKB1* rs230529 in patients >56 years old (recessive model *p* = 0.012, OR = 2.40, and 95% CI = 1.21–4.74) and *NFKB1* rs1585215 in patients with SCLC (recessive model: *p* = 0.045, OR = 14.66, and 95% CI = 1.06–203.60); *RAD52* rs7963551 in male patients (additive model: *p* = 0.046, OR = 1.49, and 95% CI = 1.01–2.22); *NFKBIA* rs2233406 (additive model: *p* = 0.029, OR = 6.73, and 95% CI = 1.22–37.16 dominant model: *p* = 0.029, OR = 6.73, 95% CI = 1.22–37.16); and *TNFRSF1A* rs4149570 in patients with lung cancer with stage III/IV cancer (dominant model: *p* = 0.050, OR = 0.26, and 95% CI = 0.07–1.00) (Table 5; Figure 2).

**TABLE 5:** Stratification analyses of Association between the seven polymorphisms and PFS in lung cancer patients.

Genes	SNPs	Subgroups	Additive		Dominant		Recessive	
			OR (95%CI)	*p* value	OR (95%CI)	*p* value	OR (95%CI)	*p* value
BRCA2	rs206118	Age (≤56)	0.56 (0.33–0.97)	0.039*	0.52 (0.27–0.97)	0.041*	0.41 (0.08–2.05)	0.279
XRCC3	rs1799794	Age (>56)	1.38 (0.93–2.04)	0.112	2.03 (1.05–3.92)	0.036*	1.17 (0.61–2.25)	0.641
NFKB1	rs230529	Age (>56)	1.36 (0.90–2.06)	0.150	0.94 (0.48–1.82)	0.854	2.40 (1.21–4.74)	0.012*
	rs1585215	SCLC	1.78 (0.72–4.41)	0.216	1.13 (0.35–3.67)	0.840	14.66 (1.06–203.6)	0.045*
RAD52	rs7963551	Male	1.49 (1.01–2.22)	0.046*	1.52 (0.96–2.43)	0.077	2.29 (0.73–7.17)	0.154
NFKBIA	rs2233406	Stage (Ⅲ/Ⅳ/ED)	6.73 (1.22–37.16)	0.029*	6.73 (1.22–37.16)	0.029*		
TNFRSF1A	rs4149570	Stage (Ⅲ/Ⅳ/ED)	0.63 (0.28–1.43)	0.271	0.26 (0.07–1.00)	0.049*	1.19 (0.30–4.68)	0.803

Additive model: comparison between minor allele subjects and major allele subjects. Dominant model: comparison between minor allele carriers and major homozygous subjects. Recessive model: comparison between major allele carriers and minor homozygous subjects. OR, odds ratio; CI, confidence interval; *p*, *p*-value for binary logistic regression analysis; Ref., reference. *p < 0.05.

**FIGURE 3:**
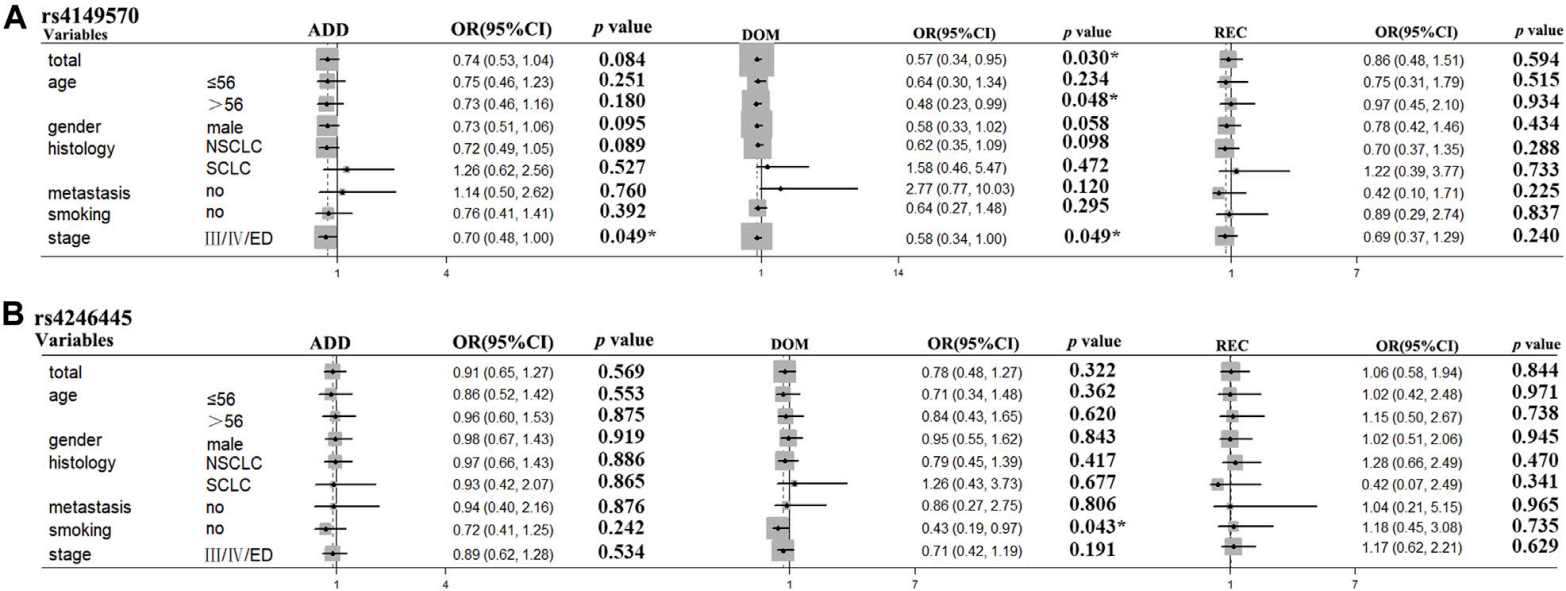
*TNFRSF1A* rs4149570 and *FASN* rs4246445 polymorphisms were significantly associated with survival times in the subgroups of patients with lung cancer treated with platinum-based chemotherapy. **(A)** *TNFRSF1A* rs4149570 polymorphism was significantly associated with OS time in patients less than 55 years of age. **(B)** *FASN* rs4246445 polymorphism was significantly associated with PFS time in patients who received platinum-based chemotherapy.

For OS stratification analyses, the results were as follows: *TNFRSF1A* rs4149570 in patients >56 years old (dominant model: *p* = 0.048, OR = 0.48, and 95% CI = 0.23–0.99); *TNFRSF1A* rs4149570 in patients with advanced stage cancer (additive model: *p* = 0.049, OR = 0.70, and 95% CI = 0.48–1.00; dominant model: *p* = 0.049, OR = 0.58, and 95% CI = 0.34–1.00); *XRCC3* rs1799794 in patients with SCLC (dominant model: *p* = 0.048, OR = 2.27, and 95% CI = 1.01–5.13); *XPC* rs2228000 in non-smokers (dominant model: *p* = 0.023, OR = 2.53, and 95% CI = 1.13–5.64); and *FASN* rs4246445 in non-smokers (dominant model: *p* = 0.043, OR = 0.43, 95% CI = 0.19–0.97) (Table 6; Figure 3).

**TABLE 6:** Stratification analyses of association between the four polymorphisms and OS in lung cancer patients.

Genes	SNPs	Subgroups	Additive		Dominant		Recessive	
			OR (95%CI)	*p* value	OR (95%CI)	*p* value	OR (95%CI)	*p* value
TNFRSF1A	rs4149570	Age (>56)	0.73 (0.46–1.16)	0.180	0.48 (0.23–0.99)	0.048*	0.97 (0.45–2.10)	0.934
		Stage (Ⅲ/Ⅳ/ED)	0.70 (0.48–1.00)	0.049*	0.58 (0.34–1.00)	0.049*	0.69 (0.37–1.29)	0.240
XRCC3	rs1799794	SCLC	2.27 (1.01–5.13)	0.048*	2.28 (0.71–7.30)	0.164	4.47 (0.97–20.71)	0.055
XPC	rs2228000	Non-smoker	1.70 (0.94–3.09)	0.081	2.53 (1.13–5.64)	0.023*	0.99 (0.27–3.63)	0.992
FASN	rs4246445	Non-smoker	0.72 (0.41–1.25)	0.242	0.43 (0.19–0.97)	0.043*	1.18 (0.45–3.08)	0.735

Additive model: comparison between minor allele subjects and major allele subjects. Dominant model: comparison between minor allele carriers and major homozygous subjects. Recessive model: comparison between major allele carriers and minor homozygous subjects. OR, odds ratio; CI, confidence interval; *p*, *p*-value for binary logistic regression analysis; Ref., reference. *p < 0.05.

Our results showed that *MAD2L2* rs746218 and *TNFRSF1A* rs4149570 were significantly associated with prognosis in patients with lung cancer who received platinum-based chemotherapy. The PFS time of patients with GG genotype (Median PFS: 3.45 (0.10–9.17) years) of *MAD2L2* rs746218 was longer than that in patients with GA or AA genotypes (Median PFS: 2.56 (0.04–11.85) years). Furthermore, OS was longer in patients with the GG genotype of *TNFRSF1A* rs4149570 than that in patients with the AA or AG genotypes. In the subgroup analysis, *BRCA2* rs206118, *XRCC3* rs1799794, *NFKB1* rs230529, *RAD52* rs7963551, *NFKB1* rs1585215, *NFKBIA* rs2233406, and *TNFRSF1A* rs4149570 were associated with the PFS time. Patients younger than 56 years old with the TT or TC genotype of rs206118 had longer PFS times than those with the CC genotype. For *XRCC3* rs1799794, the AA and AG genotypes were associated with shorter PFS times in patients >56 years old. For *NFKB1* rs230529, the AA and AG genotypes were associated with longer PFS times in patients greater than 56 years old). For *NFKB1* rs1585215, patients with squamous cell carcinoma (SCC) patients carrying the GG genotype had significantly shorter PFS times. The TT and TG genotypes of *RAD52* rs7963551 were associated with significantly shorter PFS times compared with the GG genotype in male patients. For *NFKBIA* rs2233406, the CC and CT genotypes were associated with shorter PFS times than the TT genotype in patients with stage III/IV cancer. For *TNFRSF1A* rs4149570 in patients with stage III/IV cancer, the TT and TG genotypes were associated with longer PFS times than the GG genotype. In the subgroup analyses, *TNFRSF1A* rs4149570, *XRCC3* rs1799794, *XPC* rs2228000, and *FASN* rs4246445 were significantly associated with OS. In patients older than 56 years old, the TT and TG genotypes of *TNFRSF1A* rs4149570 were associated with longer OS than that associated with the GG genotype. For *TNFRSF1A* rs4149570, the TT and TG genotypes were associated with longer OS in patients with stage III/IV cancer. For *XRCC3* rs1799794, the GG genotype was associated with longer OS in patients with SCLC. Non-smokers with the GG or GA genotypes of *XPC* rs2228000 had shorter OS than those with the AA genotype. Non-smokers with the AA or AG genotypes of *FASN* rs4246445 had longer OS than those with the GG genotype.

## Discussion

Platinum chemotherapy is one of the most important approaches for treatment of lung cancer. Platinum agents are typically used in combination with other antitumor drugs, but efficacy is limited due to resistance (Garufi et al., 2020; Yu et al., 2020). The DNA repair system contributes to platinum resistance, which influences the curative effects of chemotherapy and negatively impacts the clinical outcomes (Simon et al., 2007; Sullivan et al., 2014; Jiang et al., 2019; Peng et al., 2020). Polymorphism research has shown that gene polymorphisms affect prognosis, the folate metabolism pathway, drug transporters, and metabolic enzymes. The DNA repair system is essential for maintaining genome integrity and preventing genome instability-associated diseases, such as lung cancer (Chen et al., 2014; Anoushirvani et al., 2019; Zhao et al., 2020). Polymorphisms in DNA repair genes play a significant role in the ability to repair DNA damage. The relationship between repair gene polymorphisms and platinum chemoresistance has received a great deal of attention with regard to sensitivity of lung cancer to chemotherapy (Longhese et al., 2010; Li et al., 2018; Makovec, 2019; Schmid et al., 2020). In this study, DNA repair gene polymorphisms were studied to identify significant biomarkers for the prediction of platinum-based chemotherapy response.

In this study, we also investigated the correlations between 35 polymorphisms in 9 DNA repair genes (*XRCC3*, *BRCA2/ZAR1L*, *XPC*, *RAD52*, *MAD2L2*, *NFKB1*, *NFKBIA*, *TNFRSF1A,* and *FASN*) with platinum-based chemotherapy prognosis in 399 patients with lung cancer. A previous study showed that *XRCC3*, *BRCA2*, and *RAD52* were involved in the HR-mediated DBS repair. *XRCC3* is a *RAD51* paralog in the HR-mediated DBS repair pathway that assists *RAD51* with HR initiation (Brenneman et al., 2002). *BRCA2* is a tumor suppressor gene critical to multiple cellular processes including DNA repair, the cell cycle, and apoptosis (Cleary et al., 2020). Mutation of *BRCA2* was shown to promote tumor sensitivity towards PARP inhibitors (Farmer et al., 2005). In addition, *RAD52* has been shown to play a major role in facilitating restart of damaged replication forks (Mortensen et al., 2009). *XPC* is the main DNA damage sensor in NER, and *MAD2L2* is a controller of NHEJ-mediated DBS repair (Van Cuijk et al., 2015; Vassel et al., 2020). *NFKB1*, *NFKBIA*, *TNFRSF1A*, and *FASN* were found to be related to DNA repair, which affects tumor sensitivity to DNA-damaging agents (Chui et al., 2010; Bredel et al., 2011; Park et al., 2012; Jones and Infante, 2015). Gene variations in these genes have been reported to correlate to onset and progression of several types of tumors.

Our results showed that *MAD2L2* rs746218 and *TNFRSF1A* rs4149570 may be biomarkers for predicting the prognosis of patients with lung cancer in response to platinum-based chemotherapy. The *MAD2L2* gene, which is essential for DNA repair, localizes to uncapped telomeres and promotes the non-homologous end-joining (NHEJ)-mediated fusion of deprotected chromosome ends and genomic instability. In addition, *MAD2L2* can control DNA breaks by inhibiting 5’ end resection (Tomida et al., 2015; Dai et al., 2020). The *TNFRSF1A* gene plays a crucial role in non-small cell lung cancer growth, invasion, and metastasis (Lee et al., 2010; Fujikawa et al., 2014; Hu et al., 2019). The *MAD2L2* containing new shield complex protein plays a critical role in the choice between homologous recombination (HR) and non-homologous end-joining (NHEJ)-mediated repair. Upregulation of *MAD2L2* (also known as *MAD2B* or *REV7*) decreases DNA end resection, which increases NHEJ and chromosomal abnormalities, resulting mitotic catastrophe in PARP inhibitor treated HR-proficient cells. In addition, *MAD2L2* can also inhibit end-resection in irradiation (IR)-induced DNA double-strand breaks (DSBs) (Boersma et al., 2015; Simonetta et al., 2018; De Krijger et al., 2021). *MAD2L2* accelerates end-joining of DNA double-strand breaks in several settings, including immunoglobulin class switch recombination through ATM kinase activity (Xu et al., 2015; Batenburg et al., 2017; Noordermeer et al., 2018). Previous studies showed that *MAD2L2* promoted DNA repair activity through 53BP1 and promotes NHEJ by inhibiting 5′ end resection downstream of RIF1 protein (Ghezraoui et al., 2018; Liang et al., 2020). Both *MAD2L2* Rs746218 and *TNFRSF1A* rs4149570 are upstream transcript variants, and might affect gene expression by interacting with promoters to influence gene transcription. Therefore, *MAD2L2* rs746218 could influence the choice between HR and NHEJ by affecting the expression of *MAD2L2*. In addition, the *TNFRSF1A* gene was shown to play a crucial role in NSCLS growth, invasion, and metastasis (Lee et al., 2010; Fujikawa et al., 2014; Hu et al., 2019). However, the mechanism by which the *TNFRSF1A* gene affects prognosis associated with platinum-based chemotherapy has not been characterized. Future studies should characterize the mechanism by which *MAD2L2* rs746218 participates in the double-strand break repair pathway and the mechanism by which the *TNFRSF1A* gene contributes to platinum chemoresistance. Characterization of these mechanisms may allow for the development of new drug candidates and more effective use of combination therapies including platinum-based drugs and DNA repair regulators.

**FIGURE 4:**
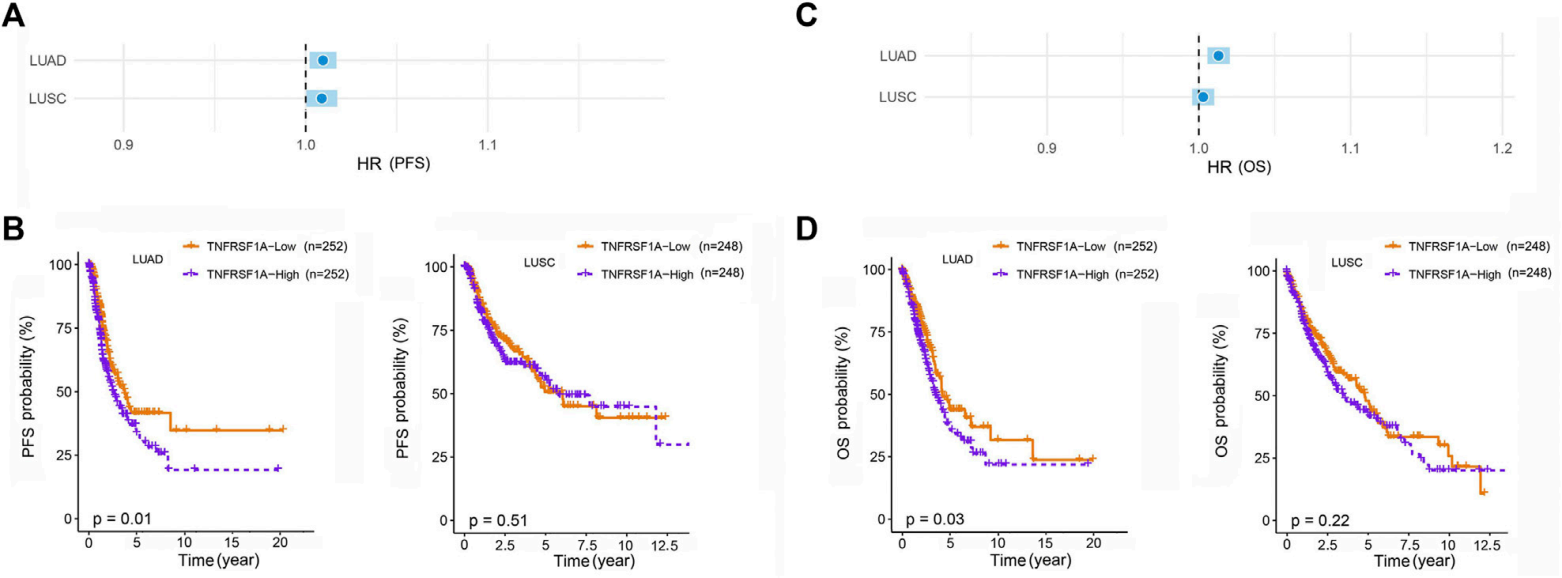
Association of the expression of *TNFRSF1A* with lung cancer prognosis in patients with LUAD (Lung adenocarcinoma) and LUSC (Lung squamous cell carcinoma). Low expression of *TNFRSF1A* in patients with LUAD was associated with significantly longer **(A,B)** progression-free survival (PFS) and overall survival **(C,D)** (OS).

Our study was subject to the following limitations. Our study was a single-center study, which limits the generalizability of the results. In addition, the small sample size resulted in a broad confidence interval for *MAD2L2* rs746218, and more samples are needed to confirm the results. Potential mechanisms by which *MAD2L2* rs746218 and *TNFRSF1A* rs4149570 impacted prognosis in patients with lung cancer who received platinum-based chemotherapy were determined using TCGA data (https://portal.gdc.cancer.gov/). This analysis showed that low expression of *TNFRSF1A* in LUAD (lung adenocarcinoma) was associated with significantly longer PFS and OS (Figure 4). However, the mechanisms of these effects require further investigation.

In summary, our study showed that *MAD2L2* rs746218 was significantly associated with platinum-based chemotherapy, and PFS and *TNFRSF1A* rs4149570 was significantly associated with OS time in patients with lung cancer treated with platinum-based chemotherapy. Polymorphisms of *MAD2L2* rs746218 and *TNFRSF1A* rs4149570 polymorphisms may be biomarkers for predicting prognosis in patients with lung cancer treated with platinum-based chemotherapy.

## Data Availability

The original contributions presented in the study are included in the article/Supplementary Materials; further inquiries can be directed to the corresponding authors.
